# Characterisation and phylogenetic analysis of the complete mitochondrial genome of *Ctenoplusia albostriata* (Lepidoptera: Noctuidae: Plusiinae)

**DOI:** 10.1080/23802359.2019.1675551

**Published:** 2019-10-11

**Authors:** Shuang Xue, Yuanchen Zhang, Shanshan Gao, Meiling Zhang

**Affiliations:** College of Biology and Food Engineering, Anyang Institute of Technology, Anyang, China

**Keywords:** Noctuidae, mitochondrial genome, *Ctenoplusia albostriata*, phylogenetic analysis

## Abstract

*Ctenoplusia albostriata* is a pest of composite plants such as *Calendula officinalis* L. and *Dahlia pinnata*. In this study, we sequenced and analyzed the complete mitochondrial genome (mitogenome) of *C. albostriata*. This mitogenome was 15,284 bp long and encoded 13 protein-coding genes (PCGs), 22 transfer RNA genes (tRNAs), and 2 ribosomal RNA unit genes (rRNAs). Gene order was conserved and was found to be identical to most other previously sequenced Noctuidae. The whole mitogenome exhibited heavy AT nucleotide bias (80.9%). Except for *cox1* started with CGA, all other PCGs started with the standard ATN codons. Most of the PCGs terminated with the stop codon TAA, whereas *cox1*, *cox2* and *nad4* end with the incomplete codon T––. Phylogenetic analysis showed that the mitogenome of *C. albostriata* was similar to *C. agnata* and *C. limbirena*, and the subfamily Plusiinae was close to Acronictinae, Heliothinae, Amphipyrinae, Noctuinae, and Hadeninae.

Plusiinae is a main subfamily in Noctuidae which comprises more than 400 species worldwide, and they are spread from the tropics to the arctic (Nomura 1998). *Ctenoplusia albostriata* (Bremer & Grey), one of the species in Plusiinae, is a pest of composite plants such as *Calendula officinalis* L. and *Dahlia pinnata* and has been recognised as a dominant defoliator of *Solidago altissima* in Japan (Uematsu 1980).

Specimens of *C. albostriata* were collected from Neixiang County, Henan Province, China (33°30′N, 111°54′E, August 2019) and were stored in Entomological Museum of Anyang Institute of Technology (Accession number AIT-E-CTE03). Total genomic DNA was extracted from tissues using DNeasy DNA Extraction kit (Qiagen, Hilden, Germany). The mitogenome sequence of *C. albostriata* was generated using Illumina HiSeq 2500 Sequencing System (Illumina, San Diego, CA). In total, 4.7 G raw reads were obtained, quality-trimmed, and assembled using MITObim v 1.7 (Hahn et al. 2013). By comparing with the homologous sequences of other Noctuidae species from GenBank, the mitogenome of *C. albostriata* was annotated using software GENEIOUS R8 (Biomatters Ltd., Auckland, New Zealand).

The complete mitogenome of *C. albostriata* is 15,284 bp in length (GenBank accession no. MN495624), containing the typical set of 13 protein-coding, 2 rRNA, and 22 tRNA genes, and 1 non-coding AT-rich region. Gene order was conserved and identical to most other previously sequenced Noctuidae (Timmermans et al. 2014; Li et al. 2015; Yao et al. 2018; Huang et al. 2019). The nucleotide composition of the mitogenome was biased toward A and T, with 80.9% of A + T content (A 39.6%, T 41.3%, C 11.3%, G 7.8%). Of the 13 PCGs, four PCGs (*nad4*, *nad4l*, *nad5*, and *nad1*) were encoded by the minority strand (N-strand) while the other nine were located on the majority strand (J-strand). Except for *cox1* started with CGA, all other PCGs started with the standard ATN codons (seven ATG, three ATT and two ATC). Most of the PCGs terminated with the stop codon TAA, whereas *cox1*, *cox2* and *nad4* end with the incomplete codon T––. Two rRNA genes (*rrnL* and *rrnS*) were located at *trnL1*/*trnV* and *trnV*/control region, respectively. The lengths of *rrnL* and *rrnS* in *C. albostriata* were 1348 and 784 bp, with the AT contents of 84.8% and 85.6%, respectively. The 22 tRNA genes vary from 64 bp (*trnY* and *trnR*) to 71 bp (*trnK* and *trnD*).

All 13 mitochondrial protein-coding genes sequences were extracted from the mitochondrial DNA sequences of 20 closely related taxa of Noctuidae, including one outgroup species from Erebidae. Phylogenetic tree was constructed through raxmlGUI 1.5 (Silvestro and Michalak 2012). Results showed that the new sequenced species *C. albostriata* got together with the same genus species *C. agnata* and *C. limbirena* with high-support value (BS = 100), and the subfamily Plusiinae was close to Acronictinae, Heliothinae, Amphipyrinae, Noctuinae, and Hadeninae (Figure 1). In conclusion, the mitogenome of *C. albostriata* is sequenced in this study and can provide essential DNA molecular data for further phylogenetic and evolutionary analysis of Noctuidae.

**Figure 1.**
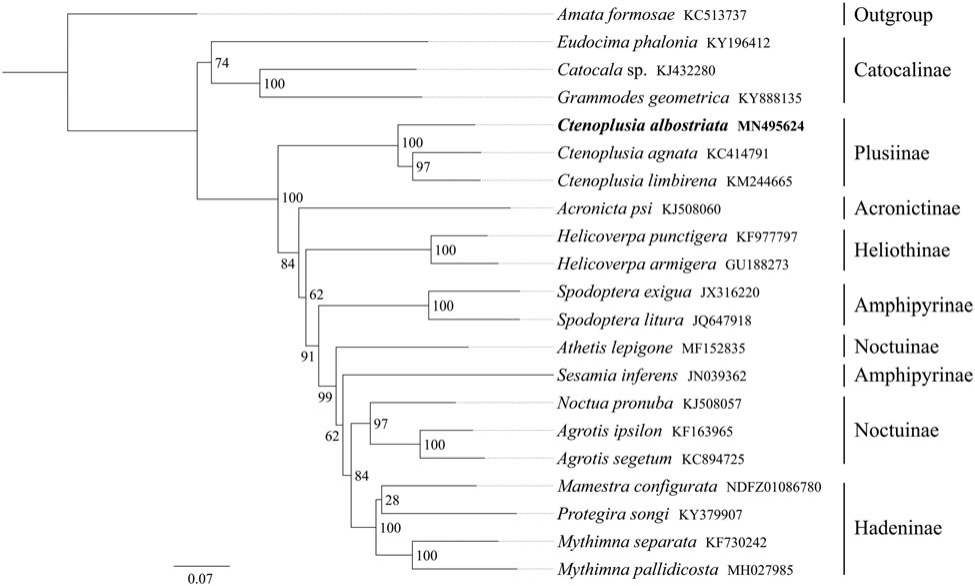
Phylogenetic relationships based on the 13 mitochondrial protein-coding genes sequences inferred from RaxML. Numbers on branches are Bootstrap support values (BS).
